# Coping with Disaster: General Practitioners’ Perspectives on the Impact of the Canterbury Earthquakes 

**DOI:** 10.1371/currents.dis.cf4c8fa61b9f4535b878c48eca87ed5d

**Published:** 2014-04-02

**Authors:** Sarb Johal, Zoe Mounsey, Robyn Tuohy, David Johnston

**Affiliations:** Joint Centre for Disaster Research, Massey University / GNS Science, Wellington, New Zealand; Joint Centre for Disaster Research, Massey University / GNS Science, Wellington, New Zealand; Joint Centre for Disaster Research, Massey University / GNS Science, Wellington, New Zealand; Joint Centre for Disaster Research, Massey University / GNS Science, Wellington, New Zealand

## Abstract

Aim – To explore the challenges for general practitioners (GPs) following the 2010/2011 Canterbury earthquakes and describe how these were met. Methods – Qualitative study using semi-structured interviews with eight GPs from the Christchurch area exploring their experiences. Results – The interviews revealed that the GPs faced a range of challenges both in the immediate aftermath of the earthquakes and in the following months. These included dealing with an increased and changed workload, and managing personal concerns. The GPs reflected on their coping behaviour and how their professional practice had changed as a result. Conclusions – All GPs reported significant increases in workload raising questions about the need for coordination of locum support. GPs often found themselves working outside their area of accustomed expertise especially in relation to patients needing financial aid. GPs identified a number of coping behaviours though some only in hindsight. Greater awareness of self-care strategies would benefit GPs responding to disasters.

## Introduction

Delivering best practice health services in the context of large-scale disaster events is a complex and challenging task. When a disaster like an earthquake strikes, the wide-ranging physical impacts also cause social and psychological disruption at community, infrastructure and service levels^1^ . The more extreme the hazardous conditions, the larger the social and emotional effects tend to be^2^ . Reactions to an extreme event such as a major earthquake have been found to vary. An extensive review^3^ ** showed that the first year after the disaster typically marks the peak of symptoms and mental health impacts. The majority in the community will experience some degree of emotional and psychological distress, which tends to remit over time without formal intervention^4^ .

Within New Zealand, local general practitioners (GPs) play a significant role in attending to the health, support and referral of patients who have been affected by a disaster. One longitudinal study^5^ found that uninjured disaster witnesses increased their number of contacts with primary care doctors by a factor of 1.55 during the first year post-disaster. GPs are well placed in the community to deal with the acute and chronic mental health issues which commonly present in the post disaster phase^6^. However, GPs face the additional challenge of caring for a disaster affected community, while also being affected by the disaster themselves. Local doctors may shoulder a large proportion of the burden of health, support and referral for the affected population both in the post-immediate phase of a disaster and further on into the recovery. Numerous studies have shown that those involved in recovery as responders can also suffer from vicarious trauma or compassion fatigue^7^^,^ 8 ^,^ 9 . GPs may find themselves being positioned by the wider community as stable, responsible, influential and helpful leaders while, in reality, they may be at times feeling as lost as any other survivor of the disaster^10^ . Previous research suggests that while 80% of primary care clinicians would be willing to assist, only 20% consider themselves well prepared to respond to a disaster^11^ .

On 22 February 2011, Christchurch New Zealand experienced a devastating earthquake, causing extensive damage and resulting in the deaths of one hundred and eighty-five people. The earthquake was part of a sequence which started in September 2010 and continued with magnitude 5+ earthquakes until December 2011. However aftershocks still occur and are felt throughout the region.

This research explores how GPs are coping with the dual challenge of personal and work demands and provides valuable information to assist in future disaster education, preparation and planning resources for GPs and the local community. Information on GPs’ experiences in this major New Zealand disaster response contributes to the on-going recovery effort and preparation/planning for future events.

## Methods

Qualitative research methodology was used to explore the GPs own experiences following the earthquakes. The data were collected between November 2012 and February 2013. This meant that a number of months had passed since the start of the earthquake sequence enabling the research to include longer term impacts and challenges. The research design used semi-structured open-ended interviews to elicit extended answers to questions about the challenges GPs have faced following the earthquakes. The rationale for the semi-structured interview format was to explore the perspective of each GP, using general questions about what they have been experiencing since the disaster.

Interviews took place with eight GPs from across the Christchurch area and included practices from different socio-economic areas (poorer to more affluent suburbs). GPs were recruited using a snowball technique in which key informants with knowledge of GP services in the area nominated GPs who were then invited to participate. The interviews were scheduled at a place and time convenient for each GP and audio-taped with permission from each participant. Five female GPs and three male GPs were interviewed covering a range of employment types from locum and salaried GPs to practice director/owner. The length of interviews ranged from 21 to 53 minutes.

The transcribed interviews were analysed and coded using a grounded theory approach^15^ . These codes were used to describe common themes that recurred during the interviews. Different members of the research team conducted the data collection and analysis. The data went through several stages of coding and theme generation to understand what the participants saw as significant and important. These themes were checked through discussions within the research team.

The study was peer reviewed and judged to be of low risk. This review was recorded on the Massey University Ethics Committee low risk database after having met their set criteria, and participants were informed accordingly.

## Results

The interviews revealed that the GPs faced a number of challenges both in the immediate aftermath of the earthquakes and in the following months. Those challenges included dealing with an increased and different workload amidst managing personal concerns. The GPs also reflected on their coping behaviour and how their professional practice changed as a result.

**Impact**

The practices experienced differing levels of impact immediately following the earthquakes from “*sudden, dramatic, phones gone, computers gone, power’s gone, liquefaction all around us, water all around us, two or three feet*” to no physical or structural damage. The GPs themselves experienced differing levels of personal impact:

“*I think it was 16 months we were out of our home…living out of a suitcase*”.

However despite these difficulties a real sense of dedication to patients was evident in the interviews:

“*we went and visited people in the community that…we were worried about*”.

One GP talked about the help they received from the local community to get the surgery running again:

“*people coming and going with boots, and shovels and wheelbarrows…helping to clear up*”.

Those practices that did not experience significant structural damage due to geographical location were affected by the earthquakes in other ways in particular high workload due to population migration:

*“lots of people moved out to live with family out here... people told us their stories, but they were so relieved to be here where it was safe we didn’t really have the stress of the really unwell and stressed out patients, but we had more of the stress of being fully booked all the time and getting numbers through*”.

Two of the GPs, either due to physical damage to the clinic or depopulation of the area, actually found themselves without a job:

“*it really affected numbers, so consequently I was laid off from work*”.

**Personal Experience**

The GPs reflected on their experiences in the weeks following the earthquakes:

“*felt you had to be strong for people*”.

“*the physical tiredness that went with the mental tiredness...just completely wiped* out”.

This ‘emotional exhaustion’ was attributed by GPs to higher workloads and sleep deprivation due to continuing aftershocks but also to the nature of the consultations:

“*my wife and I are now working standardly up to 7 o’clock at night sometimes... and a significant amount of that will be just the extra component of emotion, somatic presentation*”.

“*it tested the limits of your scope of practice, in a physical sense it was fine but the emotional gamut that you were having to deal with*”.

The increased demands also put stress on other practice staff, with some practices experiencing loss of front desk and nursing staff:

“*[it has] fallen back on nursing staff quite a bit too to try and create protocols where they can up their game, unfortunately that has put a lot of stress on them”*

*“we’ve had attrition rate from front desk staff…they’ve pulled out*”.

When these GPs experienced significant increases in workloads they seemed reluctant to ask for help or felt that it was not available:

“*there were far, far, far worse practices than us and I think, boy, they probably needed the break far more than we did*”.

"there were no locums so we just had to cover each other".

Whereas in other areas there was a perceived surfeit of support:

"I felt the 24 hour surgery was almost over staffed".

In addition to seeing patients for stress and anxiety around the earthquakes, the GPs commented that they were often asked to help with wider issues such housing and financial support:

“*that wasn’t so clear, the pathway to facilitating financial aid for patients*”.

Those GPs who were well established in their communities with good networks seemed better equipped to provide wider help. Others, particularly those new to areas or locums found it harder. One GP experienced difficulty working in a deprived area with a high refugee population with complex inter-related needs.

**Personal/work demands**

The GPs talked about facing the dual challenge of personal and work demands, especially in the first few days after the earthquakes. For many, this resulted in putting work first. However, one GP talked about the difficulty of putting her family first:

“*I was expected to go in, but I didn’t...so yeah I did feel bad about it, but my husband was so upset that he said, I can’t, you can’t put your job before your family*”.

The longer term demands, particularly working long hours, had an impact on personal relationships:

“*she was resentful, resentful at how much time I spent at work*”.

Some GPs talked about how work became an escape from personal concerns:

“*home was more stressful than work for me*”.

**Coping behaviour**

Some GPs were aware of the potential long term impact of this ‘emotional exhaustion’ and ensured that they took care of themselves:

“*I knew I needed to recharge the batteries constantly*”.

However not all GPs were as proactive, only recognising the need for self-care in hindsight. A variety of coping behaviours were identified by the GPs including peer support, exercise, socialising and taking time out. These are detailed in the table below.

**Table 1. Types of coping behaviour reported by GPs, and frequency of endorsement:** 

Coping Behaviour	Number of GPs endorsing
Getting away from Christchurch	5
Informal peer / colleague support	5
Socialising with friends	5
Work as an escape from personal difficulties	4
Exercise	3
Working part-time / reducing hours	3
TV / Games / Hobbies	2
Personal faith / Church	2
Community support	2
Formal peer support	1
Focusing on others	1

Five of the GPs commented on the importance of getting away:

“*you need to get away from it and you probably don’t realise until you get away from it how stressful it is being here*”.

“*it was amazing just totally switch off, totally relax and yeah the contrast as soon as I came back to Christchurch, like within 24 hours my stress levels were back up again*”.

GPs commented that peer support was beneficial both informally within the practice – “*we actually met, had lunch together*” and formally from the wider profession – “*being able to mix with colleagues and talk about it*”.

Physical activity was used by several GPs:

“*managed to keep up exercise which is incredibly important*”.

Socialising and keeping up existing networks was seen as important especially where, due to housing damage, people were living in temporary accommodation:

“*go out for dinner, made sure I saw friends*”.

“*a network of friendships that we had with the church*”.

Four GPs talked about how work became an escape from personal concerns:

"home was more stressful than work for me"

One of the GPs who worked part-time felt that time away from the surgery helped them to recoup their energy and focus:

“*we all work part-time and that for most of us is to make sure we don’t get burnt out*”.

One GP had reduced his surgery hours so that he could better cope with the workload and avoid burnout.

Two of the GPs mentioned empathy exhaustion or compassion fatigue – “*you are unable to sustain that level of empathy with people all of the time*” and time away was seen as beneficial to maintain compassion.

None of the GPs indicated that they had sought any professional help with dealing with the additional stress though one suggested that it might have been helpful:

“*I didn’t actually, probably again, certainly on reflection a case could have been made for that*”.

**Professional practice**

Generally, the GPs commented on how the experience had changed their professional practice. Some of these changes were practical in nature, e.g. ensuring that everyone had each others' contact details. Others focused on behaviour, with one GP talking about the ease of communicating with patients:

“*more empathic...there is a bit of a common bond when people go through similar experiences like that*”.

A clear benefit to GPs was the ability to offer patients extended consultations to discuss concerns arising from the earthquakes as well as being able to refer patients to free counselling services:

“we had counsellors here that we had sitting here…just having cups of tea with people and being available”.

The GPs commented on the support that they received both from Pegasus (primary healthcare organisation) such as water supplies and the District Health Board (DHB) in the form of information:

“the DHB were absolutely great in getting out information to us very quickly”.

One of the GPs also commented on how open patients had become, for example talking about drinking and smoking behaviours - “*I am just surprised at how frank people are and it really makes my job easier*”.

The earthquakes raised issues about disaster preparedness, for example an inability to remain in contact with colleagues, and business continuity for the GP practices. However, GPs reported having more supplies in stock after the earthquakes and more comprehensive emergency plans in place*.*

## Discussion

In line with previous research, this study demonstrates that GPs played a major role in attending to the health and wider support needs of affected populations during and after the Canterbury earthquakes of 2010-11.

**Table 2. Lessons Offered:** 

GPs played a major role in attending to the health and wider support needs of affected populations during the Canterbury earthquake sequence, often working outside their area of accustomed expertise.
All GPs reported significantly increased workloads in the disaster response phase, and through into the recovery period, resulting in increased mental and physical fatigue.
There was a high level of awareness of the risk of compassion fatigue and burnout and a high need for self-care, but this was difficult to balance against increased community needs during the disaster.
A number of effective coping strategies were reported, including time away from the affected disaster area, informal peer and colleague support, and spending time with friends.
An identified gap concerning the coordination and provision of extra staff resource for affected practices (e.g. locum support) may be exacerbated by reluctance on the part of GPs to ask for assistance.

The impact of the disaster manifested itself in different ways. For some, the effects were focused on the physical infrastructure of the practice. For others, this affected the type and volume of workload that the practice experienced, and the staff available to deal with them. It is worth noting that GPs also commented on how their helping relationship with the community they served was not uni-directional. GPs talked about how community members helped them get their practices up and running again during the first hours and days after the earthquakes.

Despite the challenges it was clear that GPs were committed to helping their patients to the best of their abilities. While attempting to provide health services to their affected communities, GPs reflected that they experienced significant mental and physical fatigue. This speaks to the issue identified previously^10^ ; even though they were affected, GPs may feel a responsibility to provide stable, responsible and helpful leadership post-disaster.

The recent patient experience report in New Zealand^12^ showed that 84 per cent of adults surveyed expressed confidence and trust in their GP. Although this is advantageous in terms of building and maintaining the doctor-patient relationship, especially in times of great strain and community uncertainty, it also perhaps places an additional burden on GPs in a disaster. The confidence and trust that patients had in their doctors and their shared experience through the earthquakes may help to explain the increased openness described by some of the GPs regarding patient health-related behaviours. Although this frankness is to be encouraged, care needs to be taken to ensure that GPs have the knowledge and referral pathways available to them if they perceive that short-term coping behaviours risk turning into potential health hazards.

GPs also talked about threats to their income through employment displacement, and the consequent disruption and uncertainty in other aspects of their lives that this brings. Furthermore, some GPs talked about dilemmas where the careful negotiated balance between personal and professional life was highlighted for them. Some GPs found the structure and role of the professional role less stressful than their personal lives. However, immersement in their professional role may come at some personal cost as the recovery draws on.

In guarding against the risks of vicarious trauma and compassion fatigue, it is important that GPs have good awareness of self-care and practices approporiate to sustain themselves through a disaster. GPs showed an appreciation of how they may be personally affected by the earthquakes, although this was talked about when reflecting on their experiences rather than at the time they were going through the response and early-recovery period. One account focusing on disaster recovery leadership and leaders^13^ states that, “*they can face burn out at the same time the needs of the community are at their peak” *(p.30). The GPs have a leadership role within their communities and as such it is important that they, “*have a ‘self-care’ plan that outlines routines and activities that support health, relationships and allots time for pleasure and leisure*” (McNaughton, 2013, p.31)^13^ . Some GPs had consciously identified a need for self-care whereas others identified beneficial behaviours only in hindsight. Peer support was identified as helpful, as well as physical activity and connecting with broader social networks. GPs also identified other strategies of being able to manage their exposure to high and/or intense workloads, either through physically getting away for a break or moving to a part-time working structure.

It is worth noting that no GPs mentioned that they had sought any professional support, though one GP reflected that they perhaps needed it. It seems important not to assume that no requests for professional support means that it is not required. Considerable stigma still exists regarding help-seeking for mental health impacts after disasters, and GPs are likely to be as affected as other groups (Figley, 1995; Spinhoven & Verschuur, 2006: North *et al*, 2002; Somers, Drinkwater & Torcello, 1997).

Large scale natural disasters can pose serious challenges for business continuity and operations of existing health care providers and systems, such as loss of staff^14^ . Although some GPs in our study described how their practices had been affected such that they found themselves working in locum positions, other GPs talked about increased workloads but a reluctance to ask for help. Indeed, these GPs talked about how other practices perhaps needed support more than they did.

There appears to be a possible gap concerning a regionally coordinated process for identifying hotspots of GP patient demand, and the deployment of adequate resources to meet this demand, e.g. locum support. This may partly be due to the reluctance of GPs to step forward to say that they may be struggling to meet demand. However, there also appears to be a wide-scale increase in service demand due to secondary stressors such as insurance issues that puts pressure on existing services requiring an enhanced response or ‘surge capacity’.

A GP working as a locum in an unfamiliar location may not know enough about local resources or services that might be available for their patients. Additionally, some GPs talked about their struggle to identify the pathway to facilitating financial aid for patients. In both cases, GPs often found themselves working outside their area of accustomed expertise – not unusual for a GP. Nevertheless, it may be wise to be forewarned of such possible extra demands in a post-disaster situation and to prepare accordingly.
